# Risk factors for drug-related gastrointestinal ulcer:a retrospective pharmacovigilance study

**DOI:** 10.3389/fphar.2026.1784428

**Published:** 2026-03-10

**Authors:** Shunlei Jiang, Meng Wang, Xia Ren, Zhenzhen Jiang, Qian Zhu, Songshan Dai, Jixu Li, Zhiqiang Zhao, Liang Han

**Affiliations:** 1 The First Clinical Medical College, Nanjing University of Chinese Medicine, Nanjing, China; 2 Department of Spleen and Stomach Diseases, Liyang Hospital of Chinese Medicine, Changzhou, China; 3 Institute of Basic Theory for Chinese Medicine, China Academy of Chinese Medical Sciences, Beijing, China; 4 Jincheng People’s Hospital, Jincheng, China; 5 Fengxian People’s Hospital, Xuzhou, China

**Keywords:** adverse events, drug-related, FAERS, gastrointestinal ulcers, pharmacovigilance

## Abstract

**Objective:**

Despite the fact that many medications have been linked to gastrointestinal ulcers, the extent to which most of these drugs contribute to such ulcers is not well understood. This study investigates the risk factors linked to gastrointestinal ulcers caused by drugs by analyzing large datasets obtained from the FDA Adverse Event Reporting System (FAERS).

**Methods:**

The data from the first quarter of 2015 to the third quarter of 2025 were analyzed using the report odds ratio (ROR), combined with single-factor, LASSO, and multivariate regression analysis methods, to thoroughly investigate the risk factors associated with drug-related gastrointestinal ulcers.

**Results:**

A total of 983 medications linked to adverse events concerning gastrointestinal ulcers were identified in this study, which included 21,191 patients. The medications most frequently linked to gastrointestinal ulcers include nonsteroidal anti-inflammatory drugs, anticoagulants, antiplatelet agents, and immunosuppressants. In the multifactorial logistic regression analysis, Sevelamer and acetylsalicylic acid emerged as the two medications most strongly associated with the highest incidence of gastrointestinal ulcer cases.

**Conclusion:**

Nonsteroidal anti-inflammatory drugs, Sevelamer, immunosuppressants, and other medications have shown a significant positive association with gastrointestinal ulcers. These findings provide hypothetical clues for pharmacovigilance; however, establishing a causal relationship requires further validation through prospective studies in populations.

## 1 Introduction

Gastrointestinal ulcers (GIUs) represent a prevalent gastrointestinal disorder with a notable incidence rate globally. Although the age-standardized incidence of the disease has dropped significantly by 40.3% since 1990, the absolute number of cases has climbed by 11.1% due to rising population growth and aging (Hao et al., 2025). This condition not only diminishes the quality of life but may also result in severe complications, including bleeding, perforation, and obstruction, thereby increasing the healthcare burden. The development of peptic ulcers is linked to various risk factors, among which medication use stands out as a significant and preventable contributor. Nonsteroidal anti-inflammatory medications (NSAIDs) are commonly acknowledged as a major contributor to the onset of ulcers (Islam et al., 2024). In specific populations, such as renal transplant recipients, the use of immunosuppressive drugs like methylprednisolone pulse therapy can further elevate the risk of ulcer formation (Kondo et al., 2023). However, existing studies primarily focus on gastrointestinal ulcers induced by single drugs, such as aspirin, and lack a systematic comparative evaluation of the severity of this risk among different drug categories (Huang et al., 2026).

The FDA Adverse Event Reporting System (FAERS) serves as an extensive database for monitoring drug safety, encompassing more than 20 million records of adverse events (AEs) (Sakaeda et al., 2013; Zhou and Hultgren, 2020). This research seeks to thoroughly identify possible drug-related factors contributing to gastrointestinal ulcers by utilizing the FAERS database. By doing so, it provides essential data support and theoretical foundations for risk identification, prevention, and control strategy development in clinical practice concerning drug-related gastrointestinal ulcers. Additionally, this research facilitates subsequent targeted studies and serves as a medication reference for clinicians.

## 2 Materials and methods

### 2.1 Data sources and data processing

The data for this study were sourced from the publicly accessible FAERS database, which is updated on a quarterly basis. The extraction period spanned from the first quarter of 2015 to the third quarter of 2025. The FAERS database utilizes the International Medical Dictionary for Regulatory Activities (MedDRA) version 27.1 for adverse event coding (Tieu and Breder, 2018). The inclusion criteria were strictly defined as follows: (1) Adverse Events: The primary adverse event of interest was “gastrointestinal ulcer,” encompassing the following MedDRA Preferred Terms (PT): “oesophageal ulcer,” “peptic ulcer,” “gastric ulcer,” “duodenal ulcer,” “gastroduodenal ulcer,” “ileal ulcer,” “intestinal ulcer,” “jejunal ulcer,” “large intestinal ulcer,” and “rectal ulcer” (PT codes: 10030201, 10034341, 10017822, 10013836, 10017886, 10021309, 10022714, 10023177, 10023799, 10038080). (2) Drug Exposure:Reports were required to contain at least one primary suspect drug (Role_cod = “PS”) with a complete and identifiable drug name. (3) Time Frame:The occurrence date of the event or the FDA receipt date had to fall between 2015Q1 and 2025Q3. The exclusion criteria were as follows: (1) Duplicate Reports:Duplicates were removed based on the FDA-assigned case number (CASEID) and primary identifier (PRIMARYID), retaining only the most recent FDA receipt version for each case. (2) Unmapped Drug Names: Records whose drug names could not be mapped to the WHO Drug Global or the Anatomical Therapeutic Chemical (ATC) classification system were excluded. Given that drug names in the raw FAERS data are stored as free text, encompassing brand names, generic names, spelling variants, abbreviations, and non-standard expressions, we implemented the following standardization workflow to facilitate classification: (1) Brand-to-Generic Mapping:Utilized the FDA-provided drug name cleaning tool to map brand names to their generic equivalents. (2) WHO Coding: Coded the cleaned generic names according to the WHO Drug Global standard. (3) ATC Mapping: Mapped the coded drugs to the ATC system at both the second level (therapeutic subgroup) and the fifth level (chemical substance). Drugs that could not be mapped to the ATC system were excluded from subsequent analyses.

### 2.2 Statistical analysis

The report odds ratio (ROR) is our main tool for signal mining, being the statistical approach most frequently utilized in disproportionality analysis. This method relies primarily on the four-row table used for the proportionality test (Table 1). A drug is deemed related to peptic ulcer disease if “a,” the number of reported cases, is greater than or equal to 3, and the lower limit of the 95% confidence interval (CI) for the ROR exceeds 1. The specific formula is shown in Table 2. It is important to note that a positive ROR signal merely indicates that the reporting proportion of the drug for ulcer events exceeds that of all other drugs in the database, suggesting a potential signal, but it does not equate to a causal risk.

**TABLE 1:** Four fold table of disproportionality measures.

Type of drug	Targeted AE	Other AEs	Total
Target drug	a	b	a + b
Other drugs	c	d	c + d
Total	a + c	b + d	a + b + c + d

**TABLE 2:** Formulas and threshold values of ROR.

Methods	Calculation formula	Algorithmic signal generation conditions
ROR	$ROR=\frac{ad}{bc}$ $SE\left(\ln ROR\right)=\sqrt{\left(\frac{1}{a}+\frac{1}{b}+\frac{1}{c}+\frac{1}{d}\right)}$ $95\%CI=e^{\ln\left(ROR\right)\pm \sqrt[{1.96}]{\left(\frac{1}{a}+\frac{1}{b}+\frac{1}{c}+\frac{1}{d}\right)}}$	95%CI (lower limit) > 1, a≥3

The FAERS database aggregates reports containing detailed patient information (age, sex, and weight). Only reports with complete datasets suitable for analytical assessment were included. For specified drugs, univariate analysis was conducted based on the following criteria: lower limit of 95% confidence interval for ROR >1, report count >100, and adjusted P-value <0.01. For drugs with P < 0.01 in univariate analysis, Least Absolute Shrinkage and Selection Operator (LASSO) logistic regression with 10-fold cross-validation was performed for feature selection. The regularization parameter (λ) was optimized using the “one-standard-error rule” (λ.1se) to select the most parsimonious model. This procedure was repeated 10 times to ensure robustness. Multivariable Logistic Regression: A multivariable logistic regression model was constructed incorporating LASSO-selected drugs and baseline patient characteristics (age, sex, weight) as independent variables. Adjusted odds ratios (ORs) with 95% confidence intervals were reported. Model Validation and Performance Evaluation: Internal validation was performed using 10-fold cross-validation, with bootstrap resampling to estimate 95% confidence intervals for performance metrics. Beyond AUC, comprehensive performance metrics including sensitivity, specificity, F1 score, and positive predictive value (PPV) were reported.

All data processing and statistical analyses were performed using the R programming environment (version 4.3.2). In addition, this study strictly adhered to the READUS-PV guidelines for standardized signal detection within the FAERS database (Fusaroli et al., 2024).

### 2.3 Ethical statement

Since the FAERS database is publicly accessible and patient records are anonymized, this research does not require ethical approval or the consent of participants.

## 3 Results

### 3.1 Baseline characteristics

**FIGURE 1:**
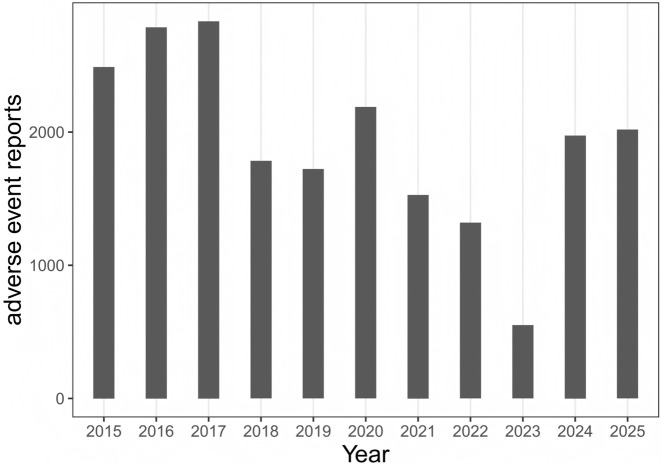
The distribution of enrolled patients by year in this study was presented in case report.

In this research, once we removed duplicate and uncertain data entries, we included 14,782,616 complete reports. By employing data mining techniques, we discovered 983 medications linked to adverse events involving gastrointestinal ulcers, which impacted a total of 21,191 patients. Using the acquired adverse event data, we performed a baseline analysis regarding gastrointestinal ulcers. Among the reporters, there were 9,582 females (45.2%), 9,051 males (42.7%), and 2,558 individuals with missing gender information (12.1%). Regarding age distribution, after excluding missing values, the predominant age group was those over 66 years old (23.3%). The primary sources of reporters were Consumers (35.6%) and Physicians (31.7%), followed by Health Professionals (11.6%) and Pharmacists (7.8%). Among ulcer-related events, duodenal ulcer accounted for 39.1%, followed by gastric ulcer (34.6%) and oesophageal ulcer (10.5%). Regarding therapeutic outcomes, 96.2% of these events were classified as serious adverse events, with 49.1% leading to hospitalization, 5.5% being life-threatening, and 8.4% resulting in death. The reports were primarily from the United States (8,918, 42.1%) and Japan (2,334, 11.0%). The specific data are presented in Table 3. Concerning the yearly distribution of reports, the average number of cases recorded annually from 2015 to the present has been 1,926 (Figure 1).

**TABLE 3:** Baseline information of drug-induced gastrointestinal ulcers.

Characteristics	Drug-related gastrointestinal ulcers (N = 21191)
Gender	
Female	9582 (45.2%)
Male	9051 (42.7%)
Unknown	2558 (12.1%)
Age	
<36	1831 (8.6%)
36–53	2050 (9.7%)
53–66	3675 (17.3%)
>66	4944 (23.3%)
Unknown	8691 (41.0%)
Reported person	
Consumer	7545 (35.6%)
Health professional	2468 (11.6%)
Pharmacist	1655 (7.8%)
Physician	6715 (31.7%)
Missing	2808 (13.3%)
Outcome	
Congenital anomaly	3 (0.0%)
Death	1788 (8.4%)
Disability	87 (0.4%)
Hospitalization	10401 (49.1%)
Life-threatening	1166 (5.5%)
Other	7746 (36.6%)
Reported Country (top 4)	
United States	8918 (42.1%)
Japan	2334 (11.0%)
Canada	1966 (9.3%)
United Kingdom	1358 (6.4%)

### 3.2 Drugs associated with gastrointestinal ulcers

**FIGURE 2:**
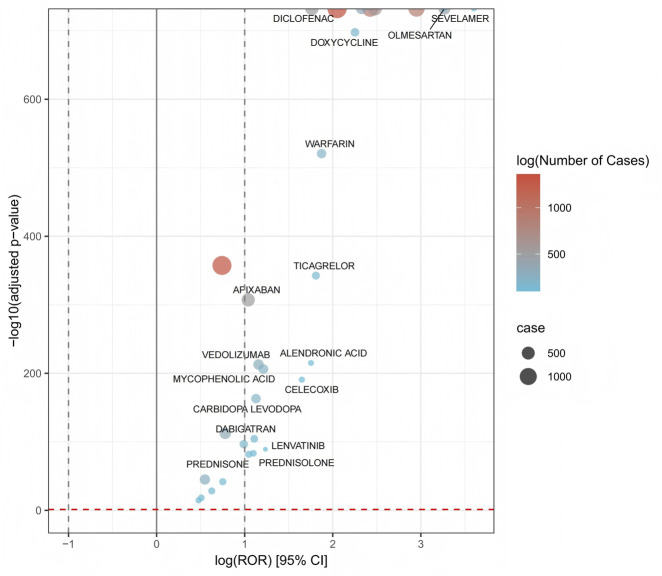
Drug-related gastrointestinal ulcer volcano chart. X-axis: log-transformed report odds ratio (ROR), indicating association strength (log [ROR] > 1 suggests positive association). Y-axis: negative log10-transformed adjusted P-value, indicating statistical significance (higher values = stronger significance). Vertical dashed lines: gray line at ROR = 1 (no association); black dashed line at log (ROR) = 1 (positive association threshold). Horizontal red dashed line: significance threshold (P = 0.05). Circle size represents number of reported cases (small = 500, large = 1000). Color scale (blue to red) represents log-transformed case count (blue: <500 cases; red: >1000 cases). Drugs in the upper right quadrant (e.g., DICLOFENAC, SEVELAMER) show the strongest positive associations.

Volcano plots were employed to effectively illustrate the correlation between peptic ulcers and the suspected drugs, as depicted in Figure 2. In this visualization, the x-axis reflects the logarithmic transformation of the ROR. When the x-axis value is positive, it signifies that there is a greater frequency of reported adverse reactions associated with the drugs in question, in comparison to reports of other types of adverse reactions. On the other hand, the y-axis is plotted as the negative logarithm of the p-value obtained from Fisher’s exact test, adjusted with the Bonferroni correction. A positive reading on the y-axis indicates that the differences observed are statistically significant. Additionally, the color coding of the points in the volcano plot is relevant, as it represents the logarithm of the total number of case reports. A deeper red hue corresponds to a higher number of reports, thereby emphasizing the drugs that have attracted substantial attention. As a result, the drugs found in the upper right corner of the plot not only indicate considerable signal intensity but also reveal statistically significant differences.

In this study, the top five drugs associated with gastric and duodenal ulcers (frequency >600) were, in descending order: rivaroxaban (ROR: 7.76, 95% CI: 7.34–8.20), adalimumab (ROR: 2.10, 95% CI: 1.99–2.22), ibuprofen (ROR: 11.30, 95% CI: 10.57–12.08), acetylsalicylic acid (ROR: 19.12, 95% CI: 17.86–20.47), and naproxen (ROR: 11.92, 95% CI: 10.98–12.94). Conversely, the drugs with the highest ROR values were, in descending order: sodium polystyrene sulfonate (ROR: 98.19, 95% CI: 64.79–148.83), acetylsalicylic acid–caffeine (ROR: 63.15, 95% CI: 30.15–132.27), sevelamer (ROR: 36.71, 95% CI: 30.17–44.68), sulindac (ROR: 34.55, 95% CI: 12.50–95.46), and olmesartan (ROR: 26.25, 95% CI: 23.78–28.97). However, it is noteworthy that the drugs with the highest ROR values were all used at relatively low frequencies. Furthermore, we classified the top 50 most frequently identified drugs in this study according to the ATC classification system. The classifications included: antineoplastic and immunomodulating agents (23/50), blood and blood forming organs (7/50), dermatologicals (5/50), nervous system (4/50), alimentary tract and metabolism (3/50), cardiovascular system (3/50), musculo-skeletal system (2/50), genito urinary system and sex hormones (1/50), sensory organs (1/50), and various (1/50) (Table 4).

**TABLE 4:** Top 50 drugs associated with AEs related to gastrointestinal ulcer.

ATC category	Drug	Number of cases	ROR	95%CI	p-value	p-adjust
Antineoplastic and immunomodulating agents	Adalimumab*	1314	2.1	1.99–2.22	2.3156E-156	2.2832E-153
	Methotrexate*	354	2.18	1.96–2.42	1.19379E-49	1.17708E-46
	Vedolizumab*	289	3.18	2.83–3.57	1.93318E-93	1.90612E-90
	Infliximab*	283	1.73	1.54–1.95	2.45843E-20	2.42402E-17
	Mycophenolic acid*	252	3.36	2.96–3.8	1.70918E-90	1.68526E-87
	Etanercept	208	0.42	0.37–0.48	5.1567E-37	5.08451E-34
	Lenalidomide	167	0.36	0.31–0.42	1.62137E-43	1.59867E-40
	Tofacitinib	159	0.9	0.77–1.05	0.207076247	1
	Rituximab	136	1.04	0.88–1.23	0.712145871	1
	Bevacizumab*	136	2.12	1.79–2.51	6.60591E-19	6.51343E-16
	Nivolumab*	134	1.43	1.21–1.7	4.02E-05	0.039637217
	Tocilizumab*	132	1.87	1.58–2.22	4.58613E-13	4.52193E-10
	Pembrolizumab*	126	1.66	1.4–1.98	1.24128E-08	1.22391E-05
	Upadacitinib*	113	1.61	1.34–1.94	4.79165E-07	0.000472456
	Secukinumab	109	0.65	0.54–0.79	1.06459E-05	0.010496854
	Risankizumab*	104	1.43	1.18–1.73	0.00032966	0.325044297
	Lenvatinib*	103	3.44	2.83–4.17	1.42838E-39	1.40838E-36
	Abatacept	99	0.92	0.75–1.11	0.404911217	1
	Ibrutinib	87	1.05	0.85–1.3	0.681681199	1
	Capecitabine*	82	1.5	1.21–1.87	0.000281381	0.277442055
	Ustekinumab	80	0.83	0.67–1.03	0.104809188	1
	Atezolizumab*	80	2.71	2.17–3.38	5.34361E-20	5.2688E-17
	Certolizumab pegol	80	0.73	0.59–0.91	0.006419753	1
Blood and blood forming organs	Rivaroxaban*	1360	7.76	7.34–8.2	0	0
	Apixaban*	533	2.83	2.6–3.08	1.7929E-134	1.7678E-131
	Clopidogrel*	354	10.25	9.21–11.39	0	0
	Warfarin*	229	6.51	5.71–7.42	2.6805E-227	2.643E-224
	Ticagrelor*	164	6.1	5.23–7.12	7.4683E-150	7.3637E-147
	Dabigatran*	153	3.03	2.59–3.56	3.11684E-46	3.0732E-43
	Edoxaban*	78	8.17	6.52–10.22	6.7545E-105	6.6599E-102
Dermatologicals	Diclofenac*	541	5.83	5.35–6.36	0	0
	Prednisolone*	124	3	2.51–3.58	5.7218E-37	5.6417E-34
	Fumaric acid	114	0.92	0.77–1.11	0.415829811	1
	Tacrolimus*	181	2.69	2.32–3.12	8.65998E-43	8.53874E-40
	Ruxolitinib	100	1.17	0.96–1.42	0.135187847	1
Nervous system	Ibuprofen*	920	11.3	10.57–12.08	0	0
	Carbidopa levodopa*	233	3.09	2.71–3.51	1.008E-71	9.93884E-69
	Paracetamol	108	0.88	0.73–1.07	0.221834699	1
	Oxybate sodium	78	0.73	0.59–0.92	0.007128862	1
Alimentary tract and metabolism	Acetylsalicylic acid*	895	19.12	17.86–20.47	0	0
	Doxycycline*	189	9.51	8.23–10.99	3.673E-304	3.6216E-301
	Ranitidine	89	0.19	0.15–0.23	5.40011E-68	5.3245E-65
Cardiovascular system	Olmesartan*	425	26.25	23.78–28.97	0	0
	Celecoxib*	114	5.2	4.32–6.26	8.73926E-84	8.61691E-81
	Hydrochlorothiazide olmesartan*	110	26.07	21.5–31.6	1.3235E-110	1.305E-107
Musculo - skeletal system	Alendronic acid*	111	5.77	4.78–6.96	1.95871E-94	1.93128E-91
	Denosumab	93	0.44	0.36–0.54	1.3305E-15	1.31187E-12
Genito urinary system and sex hormones	Naproxen*	603	11.92	10.98–12.94	0	0
Sensory organs	Prednisone*	135	2.85	2.41–3.38	2.43726E-36	2.40314E-33
Various	Sevelamer*	108	36.71	30.17–44.68	1.188E-123	1.1714E-120

*indicates drugs that are consistent with ROR positivity.

### 3.3 Risk factors for drug-related gastrointestinal ulcer

**FIGURE 3:**
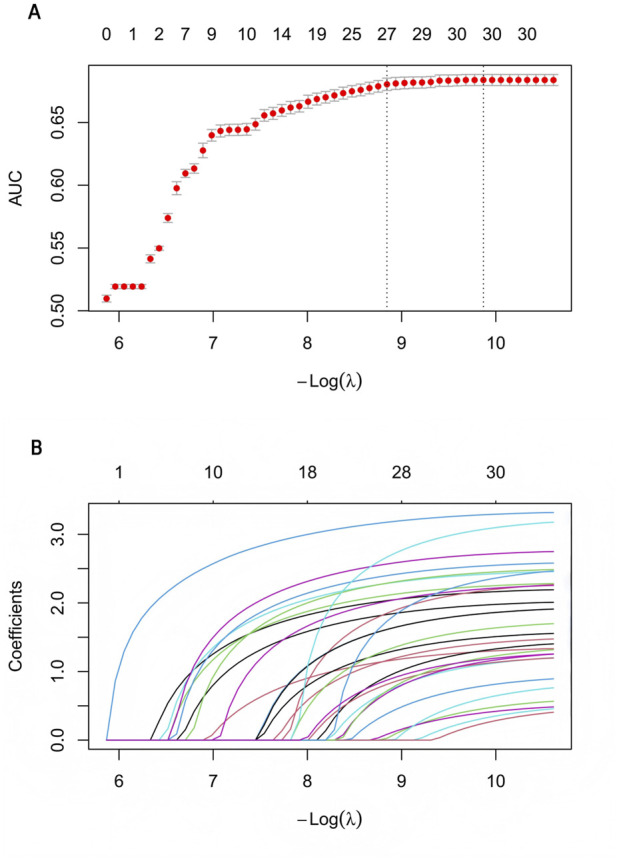
Results of the LASSO regression analysis. **(A)** AUC plot of drug-related gastrointestinal ulcers. X-axis: negative log-transformed regularization parameter (-Log [λ]), where larger values indicate weaker penalty and more features selected; Y-axis: mean AUC (Area Under the ROC Curve) from 10-fold cross-validation, with error bars representing standard error. Top numbers indicate the count of non-zero coefficients (selected features) at each λ value. Left dotted line (λ.min): regularization parameter yielding maximum cross-validated AUC (27 features); Right dotted line (λ.1se): most parsimonious model within one standard error of maximum AUC (30 features). **(B)** The Coefficients diagram of drug-related gastrointestinal ulcers. Each colored line represents the trajectory of standardized regression coefficient for an individual feature (drug) as a function of the regularization parameter. X-axis is identical to **(A)**; Y-axis: standardized regression coefficients, where positive values indicate positive association with gastrointestinal ulcer risk and negative values indicate negative association. Top numbers indicate the count of non-zero coefficients. Curves are progressively activated from left to right, with the order of emergence reflecting the relative importance of features in the prediction model.

**FIGURE 4:**
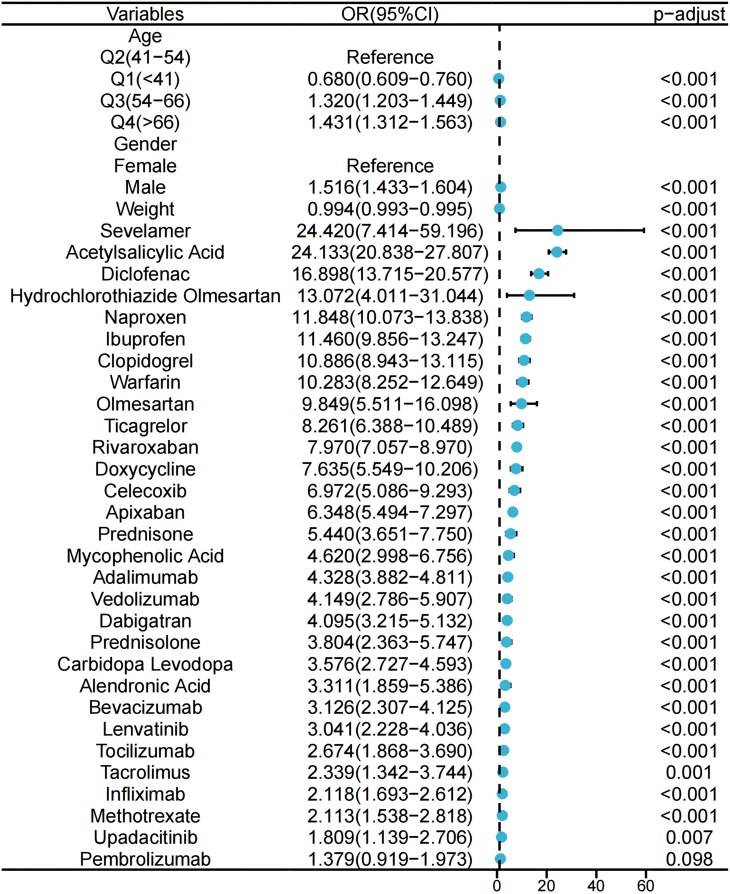
Results of the multi-factor logistic regression analysis.

**FIGURE 5:**
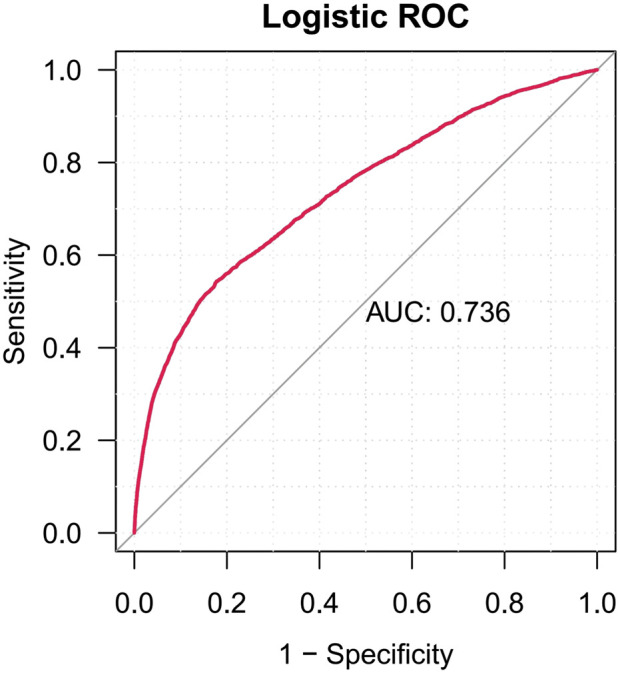
The ROC curves of drug-induced gastrointestinal ulcer.

We conducted a univariate analysis on suspected drugs with reported case numbers exceeding 100, a lower 95% CI of the ROR exceeding 1, and an adjusted p-value below 0.01. The drugs that showed p-values under 0.01 in the univariate analysis were then analyzed using LASSO regression, which led to the identification of 30 drugs (Figure 3). Subsequently, a multivariate logistic regression analysis was performed on these drugs in conjunction with patient age, gender, and other relevant information (Figure 4). The receiver operating characteristic area under the curve (ROC-AUC), which indicates the predictive accuracy of the model, was found to be 0.736 (sensitivity = 0.541, specificity = 0.824, F1 score = 0.022, PPV = 0.011) (Figure 5). Among the drugs included in the analysis, a total of 29 medications were identified as positively associated with the risk of gastrointestinal ulcers, suggesting that they may serve as independent risk factors for this condition. Sevelamer (odds ratio [OR] = 24.420, 95% CI: 7.414–59.196) and acetylsalicylic acid (OR = 24.133, 95% CI: 20.838–27.807) were identified as strong independent risk factors for gastrointestinal ulcers. Other significant medications include diclofenac, hydrochlorothiazide, olmesartan, naproxen, ibuprofen, clopidogrel, and warfarin (OR > 10). The classification of these medications follows the ATC system and is outlined as such: antineoplastic and immunomodulating agents (9/29), blood and blood forming organs (6/29), cardiovascular system (3/29), dermatologicals (3/29), nervous system (2/29), alimentary tract and metabolism (2/29), sensory organs (1/29), musculo-skeletal system (1/29), genito urinary system and sex hormones (1/29), and various (1/29). Additionally, men over the age of 54 exhibit a significantly increased risk of developing peptic ulcers. Weight gain is associated with a slight reduction in risk; however, the clinical significance of this finding is limited.

### 3.4 Interval between episodes of gastrointestinal ulceration induced by drugs

**FIGURE 6:**
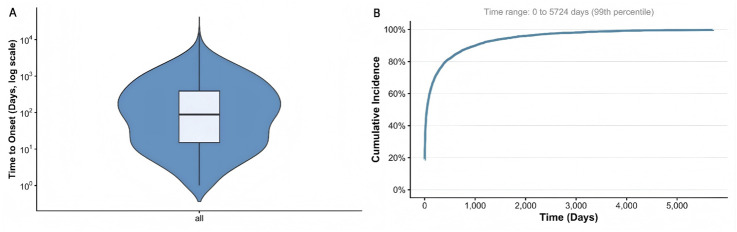
Drug-induced interval between episodes of gastrointestinal ulcer. **(A)** Violin plot of time to drug-induced gastrointestinal ulcer occurrence; **(B)** Cumulative incidence of gastrointestinal ulcers related to drug treatment.

The duration from the initiation of drug use until the development of gastrointestinal ulcers was assessed (Figure 6). The median interval for the emergence of drug-induced gastrointestinal ulcers was determined to be 63 days (IQR 330 days, Q1 9 days), with nearly 75% of documented cases arising within 339 days after the commencement of medication.

## 4 Discussion

This study systematically identifies 29 drugs that are significantly associated with gastrointestinal ulcers for the first time by analyzing data from the FAERS database between 2015 and 2025. Sevelamer (OR = 24.42) and acetylsalicylic acid (OR = 24.13) demonstrate the highest risk intensity. In addition, gastrointestinal ulcers are considerably linked to the use of anticoagulants, antiplatelet medications, immunosuppressive drugs, and biologic therapies. It must be reiterated that the conclusions of this study reflect statistical associations rather than causal relationships. The positive signals identified herein merely suggest that these drugs should be prioritized as candidates for future causal verification studies.

This study found that males aged over 54 years have a significantly increased risk of drug-induced gastrointestinal ulcers. This occurrence might be linked to the increased rates of smoking and alcohol use within this population. As age increases, the healing rate of mucosal injuries declines, rendering individuals more susceptible to the development of ulcers (Park et al., 2022). urthermore, research indicates that androgens offer weaker protective effects on the gastrointestinal mucosa compared to estrogens, thereby making the male gastrointestinal mucosa more vulnerable to drug-induced damage (Shore et al., 2021; Dean et al., 2021). The study observed a broad interquartile range (9–339 days) for the onset of adverse events, indicating that drug-induced ulcers do not follow a singular temporal pattern. Approximately 25% of events occurred within 9 days of medication initiation. These early-onset cases were primarily associated with non-steroidal anti-inflammatory drugs (NSAIDs) and other agents that exert direct mucosal injury, reflecting an acute damage mechanism. The underlying pathophysiology involves cyclooxygenase inhibition, which precipitates a sharp decline in prostaglandin synthesis, reduces mucosal blood flow, and rapidly compromises local defense mechanisms, thereby inducing ulceration within days to weeks (Eid et al., 2022). Approximately 25% of events occurred more than 339 days after drug initiation. These cases were predominantly associated with sevelamer and certain immunosuppressants, representing a chronic accumulation pattern. The ulcerogenic mechanisms of such drugs may not rely on acute cellular injury but rather involve chronic metabolic disruption or gradual immunomodulatory effects, requiring months or even years of cumulative exposure to reach the threshold for ulcer formation (Todd et al., 2024). Consequently, for drugs with a rapid onset of action and an early risk profile, monitoring efforts should be concentrated within the first month of therapy, and short-term prophylactic use of proton pump inhibitors may be considered. Clinicians should advise patients to seek prompt medical attention if they experience upper abdominal discomfort or melena during this period. For drugs with a delayed onset of action, where risk may not become apparent until several months after initiation, a long-term follow-up strategy should be implemented. Gastrointestinal symptom assessments should be performed at 3–6 months post-initiation and subsequently at 6–12-month intervals, with endoscopic screening considered when clinically indicated.

Research suggests that Svelamer (OR = 24.42) is the most critical contributor to the onset of gastrointestinal ulcers. Previous reports on sevelamer-induced ulcers have largely been confined to case reports or small case series. For instance, Ahmed Elkalashy and James Todd et al. each documented instances of colonic mucosal injury in patients with end-stage renal disease (ESRD) associated with sevelamer, suggesting that the underlying mechanism may involve the local accumulation of the drug in the intestine, leading to the formation of non-absorbable crystals (Elkalashy et al., 2025). This study, leveraging the extensive FAERS dataset, epidemiologically substantiates the systemic risk associated with the drug. Sevelamer, a non-absorbable anion exchange polymer, directly disrupts the mucus layer of the gastrointestinal mucosa, rendering it more vulnerable to acid and digestive enzymes, thus contributing to the formation of gastrointestinal ulcers (Bathobakae et al., 2024). Furthermore, constipation is one of the most prevalent side effects associated with Sevelamer (Zeng et al., 2023). The resulting delayed gastrointestinal motility extends the duration of sevelamer’s contact with the mucosa, which increases local mucosal exposure. Altered gastrointestinal motility also affects intestinal pH and the composition of contents, leading to gastrointestinal dysbiosis. Dysregulated microbiota can foster a microenvironment that promotes the initiation and progression of ulcers through mechanisms such as the activation of mucosal immunity and the production of harmful metabolites.

Nonsteroidal anti-inflammatory drugs (NSAIDs) are major contributors to the development of gastrointestinal ulcers, ranking just beneath sevelamer in terms of risk. This includes medications such as acetylsalicylic acid (OR = 24.133), diclofenac (OR = 16.898), naproxen (OR = 11.848), and ibuprofen (OR = 11.460). Motoki Kei and colleagues also conducted a study based on the FAERS database focusing on nonsteroidal anti-inflammatory drugs (NSAIDs). Their findings indicated that acetylsalicylic acid was associated with the highest incidence of gastrointestinal ulceration, whereas meloxicam was linked to the most severe cases of gastrointestinal ulceration (Kei and Uesawa, 2025). Although the risk levels ary among these medications, they share a common mechanism that entails the non-selective inhibition of cyclooxygenase-1 (COX-1), resulting in decreased production of gastroprotective prostaglandins like PGE2 (Sohail et al., 2023). Consequently, this impairment affects mucosal blood flow, the mucus-bicarbonate barrier, and epithelial repair capacity. Acetylsalicylic acid is particularly notable, ranking first among NSAIDs in terms of gastrointestinal ulcer side effects. The irreversible acetylation of platelet COX-1 by acetylsalicylic acid results in a prolonged impairment of platelet aggregation function, lasting for 7–10 days, which significantly increases the risk and severity of ulcer bleeding (Pan et al., 2025). On the other hand, Additionally, acetylsalicylic acid exhibits low solubility in the acidic environment of the stomach, allowing it to deposit and embed into the mucosa in crystalline form (Danil de Namor et al., 2022). This can cause local cellular damage and back-diffusion of hydrogen ions, leading to acute injury. Diclofenac undergoes significant enterohepatic circulation, resulting in sustained high local concentrations of the active drug in the intestinal mucosa (Zhong et al., 2016). This mechanism is critical in explaining how Diclofenac induces extensive ulceration, affecting regions from the gastroduodenal area to the small intestine. Additionally, due to Diclofenac’s highly selective inhibitory effect on cyclooxygenase-2 (COX-2), it not only suppresses inflammatory responses but also disrupts prostaglandin-dependent processes such as mucosal repair and angiogenesis, thereby hindering ulcer healing (Eid et al., 2022). In contrast, Naproxen maintains sustained and stable COX-1 inhibition through its prolonged plasma half-life, significantly extending the period during which gastric mucosal prostaglandin synthesis is insufficient and reducing the opportunity for mucosal self-repair (Nedeljković et al., 2023). Furthermore, the prolonged pharmacological action of Naproxen continuously inhibits the production of thromboxane A2 throughout the treatment duration, resulting in a marked antiplatelet aggregation effect (Katsanopoulou et al., 2025). These two mechanisms synergistically increase the cumulative risk of hemorrhagic ulcers in patients undergoing long-term Naproxen therapy. The gastrointestinal damage risk associated with Ibuprofen may be more dependent on the peak concentration effect of the drug. At high doses, Ibuprofen achieves elevated local concentrations in the stomach, where the combined effects of direct acidic irritation and systemic COX inhibition significantly elevate the risk of gastrointestinal ulcer formation (Meijuan et al., 2023).

The gastrointestinal mucsa’s integrity is preserved through efficient hemostatic processes, along with the ongoing renewal and repair of epithelial cells (Takahashi et al., 2025). Anticoagulants, such as Warfarin (OR = 10.283), Rivaroxaban (OR = 7.970), and Apixaban (OR = 6.348), as well as antiplatelet agents like Clopidogrel (OR = 10.886) and Ticagrelor (OR = 8.261), interfere with these processes, resulting in hemostatic dysfunction and impaired mucosal repair. Antiplatelet drugs, including Clopidogrel and Ticagrelor, inhibit platelet aggregation, thereby reducing the release of platelet-derived growth factor (PDGF). This inhibition increases the susceptibility of minor mucosal injuries to progress into overt bleeding and ulcers (Chung et al., 2022; Li et al., 2017). Warfarin disrupts the production of clotting factors dependent on vitamin K (II, VII, IX, and X), thereby impairing the coagulation cascade and considerably prolonging the clotting process after mucosal injury (Jiang et al., 2021). Rivaroxaban and Apixaban directly inhibit factor Xa or IIa, effectively blocking the common pathway and final steps of the coagulation cascade, which suppresses fibrin clot formation and promotes ulcer development and bleeding (Sengupta et al., 2023).

This study found that the use of immunosuppressants is a significant risk factor for gastrointestinal ulcers, including Mycophenolic Acid (OR = 4.620), Adalimumab (OR = 4.328), Vedolizumab (OR = 4.149), and Bevacizumab (OR = 3.126). Mycophenolic Acid, a purine synthesis inhibitor, exerts immunosuppressive effects by potently inhibiting the proliferation of T and B lymphocytes (Liu et al., 2025). However, it also directly inhibits the rapidly renewing gastrointestinal mucosal epithelial cells, potentially leading to atrophy of the digestive tract mucosa, impaired renewal and repair, and the induction of apoptosis in epithelial cells (Deng et al., 2022). Adalimumab, which is a monoclonal antibody targeting tumor necrosis factor-α (TNF-α), effectively inhibits intestinal inflammation; however, it might also interfere with TNF-α′s protective role in preserving the integrity of the intestinal mucosal barrier and facilitating epithelial repair (Ninnemann et al., 2022). Similarly, Vedolizumab specifically blocks the α4β7 integrin, inhibiting lymphocyte migration to the gastrointestinal tract. Although Vedolizumab effectively reduces intestinal inflammation, it may concurrently impair local immune surveillance and the maintenance of homeostasis in the intestinal mucosa, thereby affecting the microenvironment essential for intestinal ulcer healing (Arijs et al., 2018). Bevacizumab, an anti-vascular endothelial growth factor (VEGF) drug, primarily induces ulcers through the inhibition of angiogenesis, resulting in mucosal ischemia, hypoxia, and necrosis, which can trigger ulcer formation and potentially lead to gastrointestinal perforation (Sugishita et al., 2025). However, in contrast to our findings, retrospective cohort studies conducted in renal transplant populations often underestimate the gastrointestinal risks associated with immunosuppressants (Kiberd et al., 2024). This discrepancy may be attributed to the routine prophylactic co-administration of proton pump inhibitors in renal transplant recipients, which introduces confounding by indication. By employing LASSO regression for variable selection and multivariate adjustment, our study excluded the protective effect of prophylactic medication in the general population, thereby more accurately revealing the intrinsic risk associated with immunosuppressants.

This study leveraged FAERS data to identify drugs that are significantly associated with reports of gastrointestinal ulcers. However, it is essential to emphasize that these findings are hypothesis-generating rather than causally definitive. Future research should systematically pursue the following directions: (1) Mechanistic Studies: For the strong signal associated with sevelamer, animal experiments and *in vitro* studies should be conducted to investigate whether it induces ulcer formation through local mucosal irritation, dysbiosis, immune modulation, or other pathways, thereby establishing biological plausibility. (2) Population-Based Epidemiological Validation: Retrospective cohort studies utilizing electronic health records should be performed to control for confounding factors not captured by FAERS (e.g., *Helicobacter pylori* infection, prior ulcer history, drug dosage). These studies would quantify the true risk and assess dose-response relationships. (3) Cross-Database Replication: The robustness of the signal should be evaluated in independent spontaneous reporting systems such as WHO VigiBase and Japan’s JADER, assessing consistency across different populations and regions. Should these validation studies confirm the associated risks, they could facilitate the development of risk stratification models for high-risk populations, the optimization of monitoring strategies based on drug-specific temporal windows, provide valuable guidance for clinical prescribing decisions, and support the updating of drug labeling with verified risk information.

## 5 Limitations

The study’s limitations primarily stem from the inherent shortcomings of FAERS as a spontaneous reporting system. (1) Under-reporting: Certain adverse events, particularly mild or unrecognized drug reactions, may not be reported, resulting in the database capturing only a portion of the actual occurrences. (2) Reporting bias: This includes notoriety bias, wherein well-known drugs (e.g., NSAIDs) are more likely to be reported, while newly launched or less recognized drugs may be under-reported; and stimulated reporting, where media coverage or regulatory warnings can cause a temporary surge in reports for specific drugs. (3) Duplicate reports: The same patient and event may be submitted multiple times by different reporters; despite de-duplication methods recommended by the FDA, complete elimination of duplicates remains challenging. (4) Lack of denominator data: FAERS records only the number of events without the corresponding population of drug users, precluding the calculation of incidence rates or true risk. (5) Incomplete clinical information: Critical variables (e.g., duration of medication), dosage, comorbidities (e.g., *H. pylori* infection status), prior ulcer history, smoking, alcohol consumption, and other confounding factors are often missing, limiting the depth of confounding control. The impact of these unmeasured confounders on risk assessment is difficult to predict, especially in elderly populations. Elderly patients often have multiple chronic diseases, polypharmacy, and reduced renal function affecting drug clearance, which may further amplify bias in risk assessment. Without detailed clinical information, such confounding cannot be fully eliminated through conventional statistical methods. Even with variable selection techniques like LASSO, covariate adjustment remains inherently limited in this context (Noguchi et al., 2025). (6) Unclear temporal relationship: The sequence of event occurrence relative to medication initiation is often indeterminate, compromising the ability to infer causality.

This study employed a disproportionality analysis, a method that carries inherent methodological constraints; thus, its findings must be interpreted with caution (Noguchi et al., 2021; Noguchi and Yoshimura, 2024). The ROR fundamentally assesses whether a specific drug-event combination is reported disproportionately within the database. Consequently, a positive ROR signal merely reflects an association at the level of reporting and does not equate to a causal relationship. Disproportionality analysis serves as a signal detection tool, primarily for hypothesis generation rather than incidence estimation. Given these limitations, clinicians should not equate a “positive signal” directly with “ulcerogenic potential.” Instead, they should integrate the study’s findings with individual patient contexts, pharmacological mechanisms, and higher-level evidence to make comprehensive clinical decisions.

## 6 Conclusion

We conducted a comprehensive investigation of drug-induced gastrointestinal ulcers using the FAERS database, systematically identifying 29 suspect drugs that demonstrated a significant reporting association with gastrointestinal ulceration. These agents primarily encompass anticoagulants, antiplatelet drugs, immunosuppressants, and biologics. It is essential to emphasize that these findings are derived from disproportionality analysis and represent statistical associations rather than causal inferences. Future research should prioritize the validation of underlying biological mechanisms, the execution of prospective studies, and the adoption of diverse methodological strategies to further elucidate the causal relationship between drug exposure and gastrointestinal ulceration, as well as potential interventional measures.
